# Nanomedicine delivery systems remodel the immunosuppressive microenvironment of colorectal cancer: synergistic strategies and mechanisms of targeted immune checkpoint inhibitors

**DOI:** 10.3389/fimmu.2026.1882956

**Published:** 2026-06-26

**Authors:** Pengcheng Zhu, Anna Han, Chunhua Quan

**Affiliations:** 1 Central Laboratory, The Affiliated Hospital of Yanbian University, Yanji, China; 2 Jilin Xinchao Biotechnology Co., Ltd., Yanji, China

**Keywords:** colorectal cancer, drug delivery systems, immune checkpoint inhibitors, nanomedicine, targeted immunotherapy, tumor microenvironment

## Abstract

Colorectal cancer (CRC) is a highly prevalent malignancy worldwide. Traditional treatments, including surgery, chemotherapy, and radiotherapy, have limited efficacy in advanced-stage patients, therefore innovative strategies are urgently needed. Targeted immunotherapy and nanomedicine, as emerging therapeutic directions, offer new possibilities to address this therapeutic impasse. This review systematically summarizes the current application of targeted immunotherapy (immune checkpoint inhibitors, CAR-T cell therapy, and tumor vaccines) in CRC, as well as the research progress of nanomedicine (e.g., liposomes, polymeric nanoparticles, and metal nanoparticles) in drug delivery, immunomodulation, and combination therapy. By analyzing the latest research findings, this review explores the synergistic mechanisms of these two approaches, the clinical challenges, and future development directions, aiming to provide a theoretical basis and practical guidance for the precision treatment of CRC.

## Introduction

Colorectal cancer (CRC) is one of the most prevalent malignancies worldwide, with a five-year survival rate of less than 15% in patients with advanced metastases. The limitations of conventional treatments, including surgery, chemotherapy and radiotherapy, have driven researchers to explore more effective therapeutic strategies. In recent years, targeted immunotherapy, which attacks tumor cells by activating or enhancing the immune system, has achieved significant progress in various cancers. However, the efficacy of immune checkpoint inhibitors (ICIs) in CRC is significantly limited due to the inherent immunosuppressive characteristics of the tumor microenvironment (TME). CRC patients with proficient mismatch repair (pMMR) are typically insensitive to ICI treatment. Their TME is predominantly infiltrated by CD163+ macrophages and CD66+ neutrophils, and T cells exhibit an exhausted phenotype, leading to immune escape (1). Furthermore, intratumoral lactate accumulation and acidosis can impair T cell function, further weakening anti-tumor immunity (2). These findings reveal the significant challenges faced by single-agent immunotherapy in CRC.

Nanomedicine, with its unique size effects, surface modification capabilities, and multifunctionality, offers new possibilities for overcoming these therapeutic dilemmas. Nanocarriers can improve drug delivery efficiency, reduce systemic toxicity, and achieve immunomodulation. For example, a strategy based on selenium nanoparticles delivering an MDM2-targeting peptide inhibitor (Se@MI) can directly induce tumor cell apoptosis by restoring p53 function. In addition, it remodels the tumor immune microenvironment, leading to increased CD8+ T cell infiltration and inhibition of regulatory T cells (Tregs) function (3). Similarly, a triple-combination nanoscale coordination polymer (OX/GC/CQ) co-delivers prodrugs of oxaliplatin, gemcitabine, and 5-carboxy-8-hydroxyquinoline. These drugs are released specifically in the acidic TME, where they effectively induce immunogenic cell death and downregulate programmed death-ligand 1 (PD-L1) expression, thereby promoting the infiltration and activity of cytotoxic T lymphocytes (4). These studies indicate that nanotechnology can reverse the immunosuppressive microenvironment through precision delivery of immunomodulators.

This review systematically summarizes the latest research progress in this field from the perspectives of immunotherapy mechanisms, nanomedicine design, combination strategies, and clinical translation, providing a theoretical basis for the future development of more effective CRC treatment strategies.

## 1 Characteristics of the immune microenvironment in colorectal cancer

### 1.1 Infiltration of immunosuppressive cells

The TME of CRC is a heterogeneous and complex ecosystem, wherein the extensive infiltration of immunosuppressive cells forms a major barrier to both tumor immune escape and immunotherapy resistance. Among these, Tregs, myeloid-derived suppressor cells (MDSCs), and tumor-associated macrophages (TAMs) are the predominant populations. Rather than functioning in isolation, these cells cooperatively establish a sophisticated immunosuppressive network through multiple mechanisms. For instance, Tregs secrete inhibitory cytokines such as interleukin-10 (IL-10) and transforming growth factor-beta (TGF-β), directly inhibiting the proliferation and function of effector T cells. Concurrently, tumor cells and infiltrating immune cells highly express PD-L1, which binds to PD-1 on T cells to drive T cell exhaustion. TAMs, which predominantly exhibit the M2 phenotype, promote angiogenesis, tissue remodeling, and immunosuppression via IL-10, TGF-β, and VEGF. MDSCs further reinforce this suppressive milieu by producing arginase and reactive oxygen species (ROS), thereby limiting T cell activation and proliferation while depleting essential amino acids within the TME.

The proportion and functional status of these immunosuppressive cells are closely related to the prognosis of CRC patients. A high density of Treg infiltration in tumor tissue usually predicts a poor clinical prognosis, as their immunosuppressive activity hinders effective anti-tumor immune responses. Conversely, an increased density of tumor-infiltrating CD8+ cytotoxic T lymphocytes (CTLs) is significantly associated with longer disease-free survival in CRC patients, reflecting effective anti-tumor immune surveillance. Collectively, these clinical observations highlight the necessity of thoroughly elucidating the recruitment, differentiation, and functional regulatory mechanisms of key immunosuppressive cell populations. Such in-depth mechanistic understanding is critical for the development of innovative and precise immunotherapeutic strategies for CRC treatment.

Given the central role of immunosuppressive cells in CRC immune escape, targeting strategies against these cells have become an important research direction for enhancing the efficacy of immunotherapy. Current research hotspots include depleting Tregs and reprogramming TAMs from a pro-tumor M2 phenotype to an anti-tumor M1 phenotype. Nanodrug delivery systems (NDDS) provide powerful tools for implementing these strategies. By loading specific inhibitors or immune agonists into nanoparticles, drugs can be targeted delivered to specific immune cell subsets within the TME, thereby reducing systemic toxicity. A study successfully constructed self-assembled traditional Chinese medicine nanodrugs to reprogram TAMs from the M2 to M1 phenotype and promote dendritic cell (DC) maturation, enhancing anti-tumor immune responses (5). Furthermore, biodegradable nanocarriers such as PLGA are also widely studied for delivering immunomodulators to target and regulate the suppressive myeloid cell network in the TME, thereby enhancing the efficacy of ICIs (6). These studies indicate that precisely remodeling the immunosuppressive cells using nanotechnology is a promising strategic direction for overcoming CRC immunotherapy resistance and improving treatment response rates.

### 1.2 Expression patterns of immune checkpoint molecules

The expression patterns of immune checkpoint molecules in CRC exhibit significant heterogeneity, influenced by complex factors such as tumor mutational burden (TMB), microsatellite instability (MSI) status, and epigenetic regulation. Common immune checkpoint molecules include PD-1 and its ligand PD-L1, cytotoxic T-lymphocyte-associated protein 4 (CTLA-4), lymphocyte activation gene 3 (LAG-3), and T-cell immunoglobulin and mucin domain-containing molecule 3 (TIM-3) (7, 8). Studies show that in the TME of CRC, tumor-infiltrating CD4+ and CD8+ T cells highly express PD-1, TIM-3, and T cell immunoreceptor with Ig and ITIM domains (TIGIT). Correspondingly, tumor cells express their ligands, including PD-L1, CD66a, and CD155. The interaction of these ligand-receptor axes may inhibit T cell function in colorectal cancer TME (9). Furthermore, compared to microsatellite stability (MSS)-type tumors, microsatellite instability-high (MSI-H) CRC, due to its deficient DNA mismatch repair (dMMR) status, generates a large number of tumor-specific neoantigens, triggering a stronger anti-tumor immune response accompanied by the upregulation of immune checkpoint molecules (10, 11). This provides a molecular basis for the application of ICIs.

MSI-H/dMMR CRC, characterized by a mutational burden, exhibits a “hot” tumor phenotype with abundant tumor-infiltrating lymphocytes (TILs) and high expression of immune checkpoint molecules, making these patients highly responsive to PD-1 inhibitors such as pembrolizumab (12, 13). Key clinical trials like KEYNOTE-177 have confirmed that pembrolizumab, as a first-line treatment, significantly prolongs progression-free survival in patients with MSI-H/dMMR metastatic CRC and has become a standard regimen (14). However, the majority of CRC cases, MSS-type tumors, exhibit poor response to single-agent PD-1/PD-L1 inhibitor therapy, an approach not confirmed to be effective for this subtype (10) (15). Studies show that the objective response rate to PD-1 inhibitors in MSS-type CRC patients is only about 3%, far lower than the 43% in MSI-H patients (16). Therefore, converting “cold” tumors into “hot” tumors to improve the efficacy of immunotherapy in MSS-type patients is a current research focus.

Combination blockade of multiple immune checkpoints or integration with other immunomodulators has become an important strategy to improve immune responses in MSS-type CRC. Although such dual checkpoint blockade (e.g., nivolumab plus ipilimumab) has demonstrated synergistic effects and durable clinical benefits in MSI-H/dMMR CRC patients (17–19), its application in MSS-type CRC is still being explored. Beyond dual ICIs, combining ICIs with radiotherapy, chemotherapy, or other immunomodulators aims to remodel the TME, enhance immune cell infiltration and cytotoxic function, and may offer new avenues to overcome immunotherapy resistance in MSS-type CRC (20, 21). Collectively, these findings suggest that precise combination therapy strategies based on the TME hold promise for breaking through the immunotherapy barrier in MSS-type CRC.

### 1.3 Impairment of tumor antigen presentation and T cell priming

CRC cells interfere with effective antigen presentation through various mechanisms, thereby hindering the initial activation of T cells, leading to poor immunotherapy response. Tumor cells can evade immune surveillance by downregulating the expression of major histocompatibility complex class I (MHC-I) molecules. More importantly, CRC cells can actively disrupt DC function by secreting vesicles such as exosomes. Extracellular vesicles including exosomes derived from tumor cells act as key messengers between tumor and host cells in the tumor microenvironment, and mediate the communication network in the colorectal cancer microenvironment which contributes to immune tolerance and immune escape (22). Furthermore, intrinsic molecular changes in tumor cells directly impact DC migration and antigen presentation. The study of Wangmo et al. demonstrated that the deletion of atypical chemokine receptor 4 (ACKR4) in tumor cells restricts the migration of DCs to tumor-draining lymph nodes, thereby impairing the process of antigen presentation to T cells (23). Low expression of neoantigens leads to insufficient T cell priming, which forms the basis of early immune escape in CRC (24).

Hypoxic and acidic conditions in the TME exacerbate the antigen presentation disorder. In CRC, the DNA damage response induced by radiotherapy upregulates the expression of CD47 and PD-L1 via the ATR pathway (25). CD47 binding to signal regulatory protein α (SIRPα) directly inhibits macrophage phagocytosis, limiting antigen cross-presentation. Simultaneously, upregulated PD-L1 binds to PD-1 on T cells, inhibiting T cell activation. This adaptive immune resistance limits the systemic anti-tumor immune response induced by radiotherapy. Additionally, excessive uptake of glutamine by tumor cells depletes this substance in the TME, impairing DC maturation and antigen presentation ability, and disrupting T cell priming (26).

**Figure 1:**
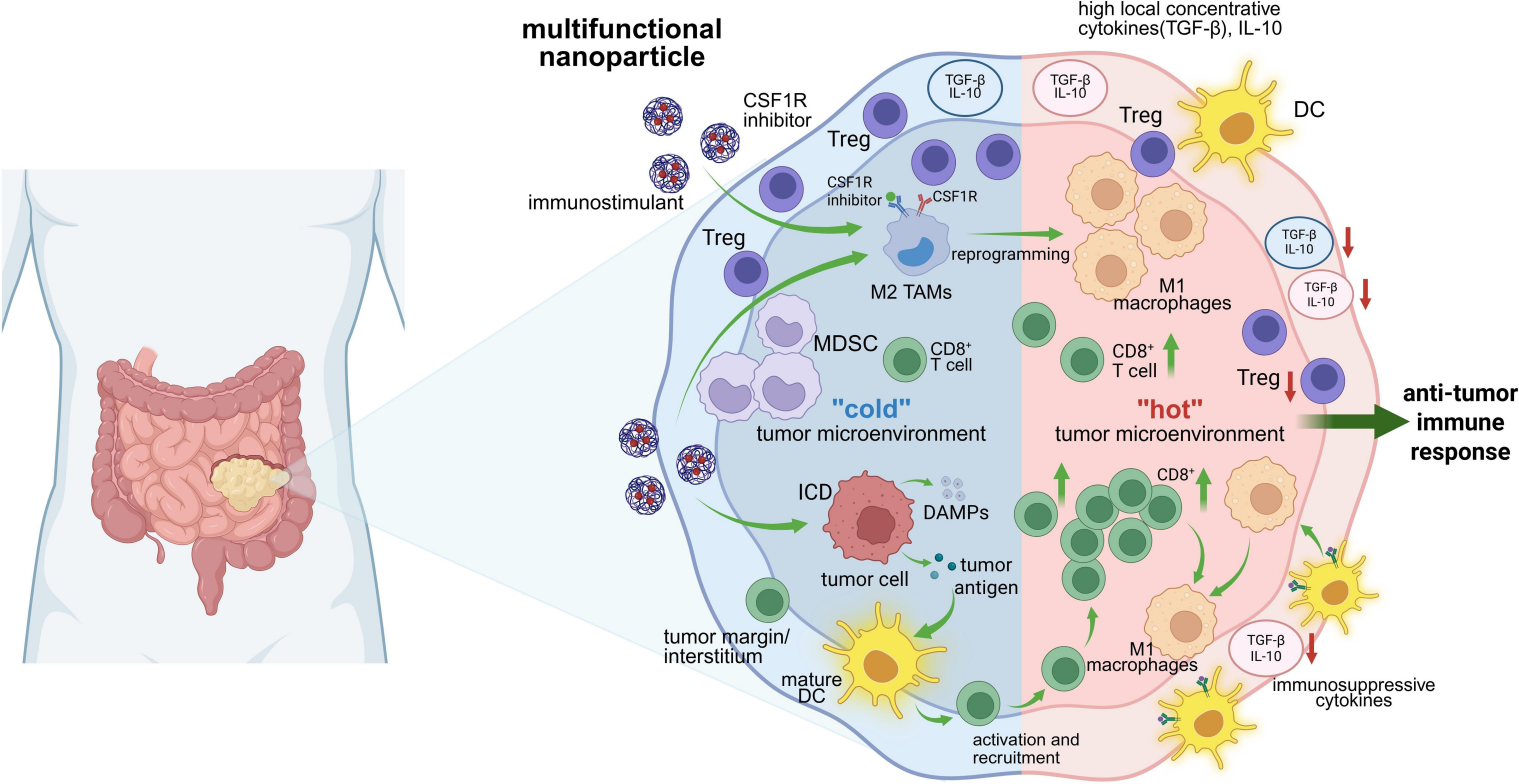
Multimodal imaging-guided integrated platform for nanomedicine. The nanoplatform integrates fluorescence (NIR-II), MRI, and photoacoustic imaging to guide treatment. Upon tumor microenvironment-responsive release, the platform delivers mild photothermal therapy, chemotherapy, and anti-PD-L1 immunotherapy, inducing immunogenic cell death, dendritic cell maturation, and CD8+ T cell activation. (Image was created with BioRender.com).

To overcome these obstacles, nanomedicine shows great potential for enhancing antigen cross-presentation and initiating specific T cell immunity. Nanocarriers can efficiently and precisely deliver tumor antigens or immune adjuvants to DCs, thereby improving antigen presentation efficiency. Platforms based on lipid nanoparticles (LNPs), such as liposomes, exosomes, and solid lip nanoparticles, are widely used for targeted delivery due to their good biocompatibility and drug-loading capacity (27, 28). These nanosystems can accumulate at the tumor site through passive targeting (enhanced permeability and retention effect) or active targeting (e.g., surface modification with antibodies, peptides, or aptamers). The study by Zu et al. developed an oral core-shell nanodrug, whose core contains a sonosensitizer and immune adjuvant R837. Nanomedicine-mediated sonodynamic therapy can induce immunogenic cell death in colorectal tumor cells, and the generated neoantigens can act as an effective *in situ* vaccine in the presence of R837 (29). Furthermore, nanomedicines can indirectly enhance DC function by regulating tumor metabolism. Studies have shown that a purpurin-copper coordination nanoplatform can inhibit glutaminase, and reprogram glutamine metabolism, thereby promoting DC maturation and T cell priming (30). These studies collectively indicate that targeted intervention of DCs through nanotechnology is an effective way to break the barriers of antigen presentation and T cell priming in CRC (**Figure 1**).

## 2 Application of targeted immunotherapy in colorectal cancer

### 2.1 Immune checkpoint inhibitors

The application of ICIs in CRC treatment marks the era of precision immunotherapy, while its efficacy is dependent on the molecular subtype of the tumor. Pembrolizumab and nivolumab, as PD-1 inhibitors, have been approved by the U.S. Food and Drug Administration (FDA) for the treatment of patients with MSI-H or dMMR metastatic CRC (mCRC) (10) (31). These drugs demonstrate significant anti-tumor activity in MSI-H/dMMR patients. However, for patients with MSS or proficient mismatch repair (pMMR) tumors, which constitute the vast majority of mCRC, the efficacy of single-agent ICIs is extremely poor (31, 32). To overcome resistance in MSS-type CRC, various combination therapy strategies are under active clinical investigation. Studies show that nivolumab combined with ipilimumab yields superior efficacy to monotherapy in MSI-H/dMMR mCRC patients, with an ORR of up to 54% and a disease control rate (DCR) of 82% (33). Furthermore, this dual immune checkpoint blockade strategy has also shown tumor growth inhibition in preclinical models of MSS-type CRC, with mechanisms potentially involving the inhibition of immunosuppressive checkpoints and the phenotypic alteration of TILs (34). Another important class of combination strategies is the addition of chemotherapy to ICIs. Preliminary clinical results indicate that combining immunotherapy with multiple treatment modalities has yielded encouraging outcomes in CRC. Moreover, the addition of agents with distinct mechanisms may improve efficacy in MSS/pMMR CRC (32, 35).

Despite the breakthrough progress of ICIs in MSI-H/dMMR CRC, primary and acquired resistance remain significant clinical challenges. After PD-1/CTLA-4 double inhibitor treatment, only TIM-3 expression is upregulated on tumor-infiltrating CD8+ T cells, while LAG-3 expression is significantly decreased; LAG-3 and TIM-3 are both actionable immune checkpoint targets for CRC treatment (7, 34). The immunosuppressive function of the tumor microenvironment is one of the factors contributing to poor treatment response in CRC patients. Studies have shown that mucinous histology is significantly associated with acquired resistance to ICIs in dMMR/MSI-H mCRC patients, and their TME may be more immunosuppressive (36). It is crucial to identify these complex resistance mechanisms for developing novel combination strategies and biomarkers to overcome resistance.

### 2.2 CAR-T cell therapy

CAR-T cell therapy for CRC has explored multiple targets, including carcinoembryonic antigen (CEA), human epidermal growth factor receptor 2 (HER2), epidermal growth factor receptor (EGFR), and guanylyl cyclase C (GUCY2C) (37, 38). These targets are overexpressed to varying degrees on CRC cells, providing potential recognition sites for CAR-T cells. Unfortunately, the inherent physical barriers and immunosuppressive microenvironment of solid tumors severely limit the actual efficacy of this therapy (39). The ineffectiveness of anti-CEACAM5 CAR-T cell therapy for CRC has been attributed to the thick glycocalyx on CRC cells and the poor accessibility of CEACAM5 (40). In addition, immunosuppressive cells (e.g., MDSCs and TAMs) together with high levels of immunosuppressive factors in the TME create a “cold” immune environment that is unfavorable for CAR-T cell survival and function. Tumor heterogeneity, along with physical and immunosuppressive barriers, limits CAR-T cell efficacy (41).

**Figure 2:**
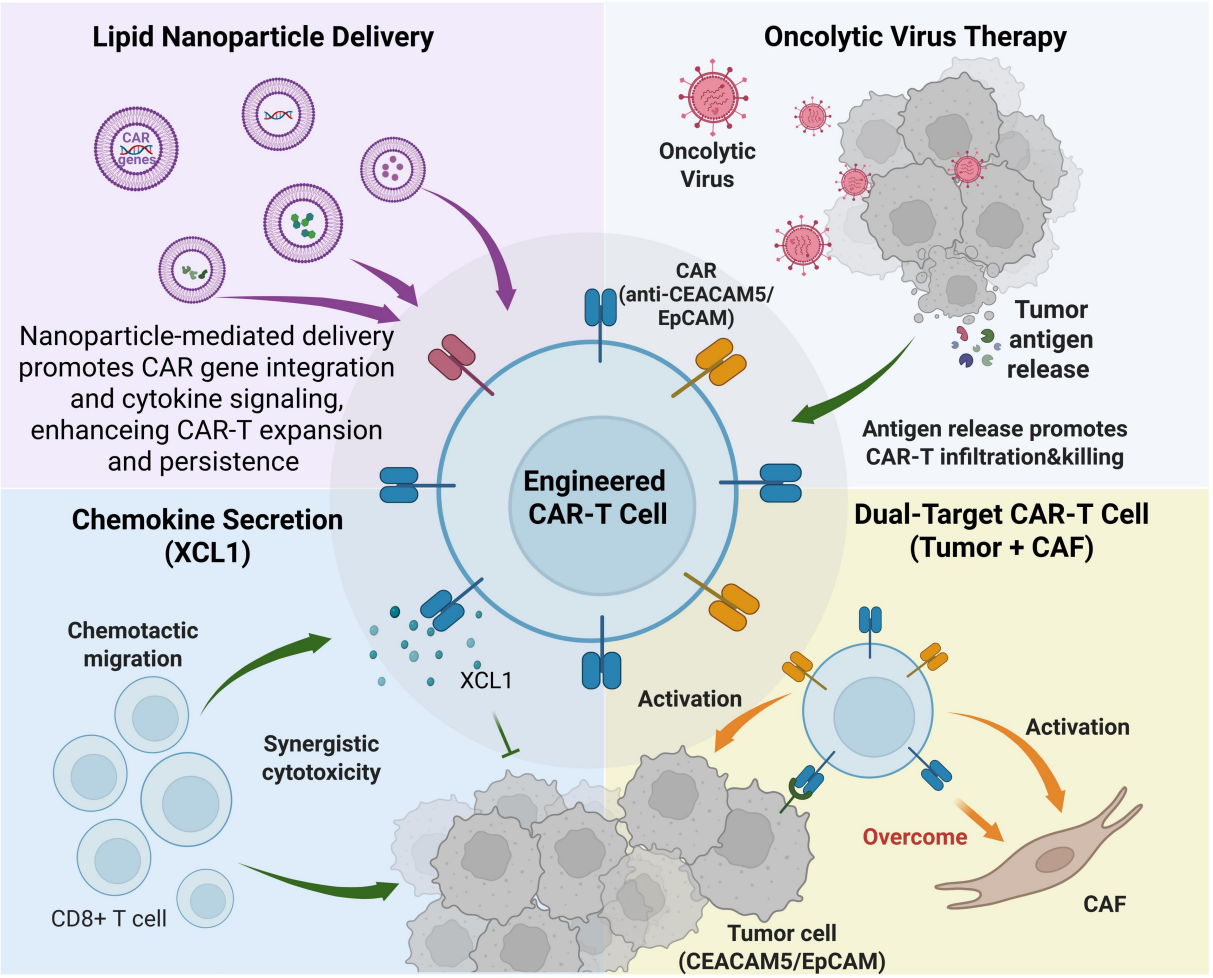
Nanoengineering strategies based on CAR-T cells enhance solid tumor therapy. (1) Nanoparticle-mediated delivery (e.g., lipid nanoparticles) of CAR genes or cytokines enhances in vivo expansion and persistence of CAR-T cells. (2) Combined with oncolytic viruses, which selectively infect and lyse tumor cells to release tumor antigens and enhance the infiltration and killing of CAR-T cells. (3) Dual-targeting CAR-T cells overcome the physical barrier by targeting both tumor cells and tumor stromal components such as cancer-associated fibroblasts (CAFs). (4) Cytokine secreting CAR-T cells, such as XCL1, recruit and activate endogenous CD8+ T cells to form synergistic anti-tumor immunity. (Image was created with BioRender.com).

To overcome these obstacles, researchers are engineering CAR-T cells to enhance their tumor homing ability and functional persistence within the TME. Strategies to improve the efficacy of CAR-T cells for colorectal cancer include engineering expression of immunostimulatory molecules, local administration, bispecific T cell engagers and combinatorial target antigen recognition (38). More importantly, combining NDDS offers new strategies for improving CAR-T cell therapy. Combination of CAR-T cell therapy with immune checkpoint inhibitors represents a promising strategy to enhance its efficacy in CRC (**Figure 2**).

### 2.3 Tumor vaccines

Tumor vaccines represent an important research direction in the field of CRC immunotherapy. Based on antigen source and delivery method, CRC tumor vaccines can be classified into personalized neoantigen-based vaccines, DC-based vaccines, and nanocarrier-based delivery systems.

A preliminary clinical study in patients with MSS-type metastatic CRC showed that personalized neoantigen vaccine therapy is safe and feasible, inducing neoantigen-specific immune responses and prolonging progression-free survival (PFS) in some patients (42). Furthermore, circular RNA (circRNA) has been discovered as an alternative source of neoantigens. Peptides encoded by circRNA can effectively activate CD8+ T cells and specifically kill patient-derived organoids, providing new vaccine targets for CRC with low mutational burden (43).

DC vaccines represent another important form of vaccine. DCs, as the most potent antigen-presenting cells, are loaded with tumor-specific antigens (e.g., tumor lysates, specific peptides, or whole tumor cells) ex vivo and then infused back into the patient to activate antigen-specific T cells. Mucin 1 (MUC1), a tumor-associated antigen highly expressed in CRC, is an important target for DC vaccines. Studies show that a CRC stem cell (CCSC) vaccine loaded with MUC1 significantly activates NK cells, T cells, and B cells in mouse models and inhibits tumor growth by targeting and killing CCSCs. Neutralizing MUC1 antibodies significantly weaken its anti-tumor effect, confirming the key role of MUC1 in the immunogenicity of the CCSC vaccine (44, 45). The first DC vaccine, Sipuleucel-T, has been approved for prostate cancer, but clinical translation of DC vaccines in CRC is still under exploration. Cancer vaccines are considered more suitable as an adjuvant to standard treatment, especially in combination with immune checkpoint inhibitors (ICIs) for patients with microsatellite-stable colorectal cancer (46).

The development of nanocarrier technology offers new solutions for the delivery of tumor vaccines. Nanocarriers can encapsulate both antigens and immune adjuvants simultaneously, achieving co-delivery. This design protects antigens and adjuvants from *in vivo* degradation and improves their lymph node targeting efficiency (47). Moreover, it can promote the uptake and maturation of antigen-presenting cells, thereby enhancing the intensity and durability of the immune response. Furthermore, a study utilized succinylated PEI-9 nanocarriers to deliver a guanylin gene driven by the MUC1 promoter, achieving specific anti-tumor effects in CRC cells, demonstrating the potential of nanosystems in precise delivery and targeted gene therapy for CRC (48).

## 3 Design strategies of nanomedicine in colorectal cancer treatment

In the treatment of CRC, nanomedicine design strategies primarily revolve around several core platforms, including liposomes, polymer nanoparticles, and metal nanoparticles, each demonstrating unique advantages and limitations in drug delivery and immunomodulation.

### 3.1 Liposomal nanomedicines

Liposomes are classic nanodrug delivery systems, showing significant advantages in CRC treatment. Their core mechanism involves encapsulating chemotherapeutic agents or immunomodulators, effectively prolonging the drug’s *in vivo* half-life, and achieving passive targeting via the enhanced permeability and retention (EPR) effect characteristic of tumor tissues. Studies show that co-encapsulating irinotecan (CPT-11) and thalidomide in liposomes can effectively inhibit tumor growth while alleviating the delayed diarrhea induced by free drugs (49). Additionally, multiple studies have confirmed that oxaliplatin, as a first-line chemotherapeutic agent for CRC, exhibits enhanced therapeutic efficacy and reduced adverse effects when delivered via liposomes (50). Building on this, the combination with targeted ligands such as anti-EGFR antibodies enables active targeting, further enhancing drug accumulation efficiency at CRC lesions (51). Similarly, liposomes modified with hyaluronic acid (HA) co-loaded with 5-fluorouracil (5-FU) and cannabidiol (CBD) achieve precise drug delivery by targeting the CD44 receptor overexpressed on CRC cells, significantly enhancing anti-tumor effects and reducing systemic toxicity (52).

Although the successful application of liposomal irinotecan (Onivyde) has provided valuable insights for the development of liposomal drugs for the treatment of CRC, the heterogeneity of the EPR effect in human tumors significantly limits its widespread application (53). Differences in tumor vascular permeability and interstitial pressure among patients lead to uneven distribution of nanomedicines within the tumor, affecting treatment efficacy. To overcome this limitation, researchers have explored various strategies. For example, modifying Camptothesome (a vesicular nanomedicine formulated with sphingosine-conjugated camptothecin) with tumor-penetrating peptides (e.g., LinTT1) initiates transcytosis by binding to the p32 protein overexpressed on tumor cells, achieving deep tumor penetration and significantly improving the anti-tumor effect of camptothecin in CRC xenograft models (54). Furthermore, innovative strategies include a multi-level delivery system formed by embedding liposomes into mesoporous silica microparticles, which protects drugs from degradation and control release, as well as the development of biomimetic magnetic liposomes loaded with oxaliplatin to enhance biocompatibility (55, 56). These studies collectively indicate that optimizing liposome design holds promise for overcoming the limitations of the EPR effect and advancing the clinical application of liposomal nanomedicines in precision CRC therapy.

### 3.2 Polymeric nanoparticles

Polymeric nanoparticles, particularly nanocarriers prepared from biodegradable polymers such as PLGA and polycaprolactone (PCL), show great potential in targeted immunotherapy and drug delivery for CRC. The core advantages of these nanoparticles lie in their ability to control drug release kinetics, improve the solubility of chemotherapeutic drugs, prolong colonic retention and achieve targeted delivery of anticancer drugs (57). Additionally, the physicochemical properties of the nanoparticles can be optimized by modulating the molecular weight and composition of the polymer. Controlling the particle size within the 50–200 nm range and adjusting their surface charge are crucial for enhancing nanoparticle penetration into tumor tissue and uptake by tumor cells (58).

PLGA nanoparticles have been used for the co-delivery of chemotherapeutic and immunotherapeutic drugs to achieve synergistic anti-tumor effects in CRC treatment (59, 60). A study designed multifunctional targeted polymeric nanoparticles co-encapsulating paclitaxel, the anti-PD-L1 antibody atezolizumab, and the anti-VEGF antibody bevacizumab for triple combination therapy in CRC. This nanoparticulate system demonstrated efficient tumor-targeted co-delivery and good therapeutic efficacy in a CRC mouse model (61). Furthermore, polymeric nanoparticles also show advantages in delivering natural products and overcoming chemoresistance (62). Co-loading beta-carotene and 5-FU into polymeric nanoparticles may overcome 5-FU chemoresistance in p53-deficient CRC cells by inducing cell cycle arrest and apoptosis, thereby enhancing therapeutic efficacy (63). These studies demonstrate that polymeric nanoparticles, as a versatile and tunable drug delivery platform, hold broad application prospects in targeted therapy for CRC (64). However, the application of polymer nanoparticles in CRC also faces challenges. The scalability and stability of their preparation processes, inter-batch consistency, and the long-term effects of *in vivo* degradation products remain significant obstacles to clinical translation.

### 3.3 Metal nanoparticles

Metal nanomaterials (such as gold nanoparticles and iron oxide nanoparticles) exhibit unique optical, magnetic, or catalytic properties in the treatment of CRC. Studies show that synthesized branched gold nanoparticles exhibit high photothermal conversion efficiency under 808 nm laser irradiation, effectively inducing membrane rupture and thermal ablation in CRC cells (65). Additionally, superparamagnetic iron oxide nanoparticles can stably generate heat when simulating the physiological environment of the colon, with a temperature increase of over 5 °C within 10 minutes, showing potential as magnetic hyperthermia agents (66). These physical treatment methods can not only directly kill tumor cells but also induce ICD, release tumor-associated antigens and activate the immune system, creating favorable conditions for subsequent immunotherapy.

To further enhance therapeutic efficacy, metal nanoparticles are often used as multifunctional platforms, conjugating immunomodulatory molecules to their surface to achieve synergy between physical therapy and immunotherapy (67). Furthermore, modifying guanylyl cyclase-targeting monoclonal antibodies onto the surface of gold nanoparticles can actively target CRC cells, improving the specificity and efficiency of drug delivery, offering possibilities for photodynamic therapy (68). These surface-engineered nanoplatforms can precisely focus physical energy on the tumor site while locally releasing immunomodulatory molecules, remodeling the TME to achieve synergistic therapeutic effects.

However, the long-term biosafety of metal nanoparticles remains a major concern. Unmodified citrate-coated gold nanoparticles, after 12 weeks of exposure in a rat model, significantly increased the levels of TNF-α and IL-6 in brain tissue, suggesting they may induce delayed neuroinflammation (69). In contrast, PEG-modified gold nanoparticles and their conjugates with CRC-targeting peptides showed no significant inflammatory or apoptotic effects at the same time point, indicating that PEGylation can effectively reduce the risk of neurotoxicity. Nevertheless, repeated administration may induce the production of anti-PEG antibodies, accelerating drug clearance and triggering allergic reactions, which has become a common obstacle for all PEGylated nanomedicines. To overcome this problem, researchers are exploring PEG alternatives, such as polymer nanoparticles coated with amphiphilic natural starch or optimized PEG chain conformation to reduce non-specific interactions, but these alternative strategies are still in early validation stages (70, 71).

Different nano-delivery systems have shown their own advantages and limitations in the treatment of colorectal cancer (**Table 1**). No single nanoplatform fits all colorectal cancer patients. Rational choices for clinical translation must be based on the specific treatment scenario. In the future, only by accurately matching the physicochemical properties of nanoplatforms with the pathophysiological characteristics of specific patient groups can the clinical value of individualized nano-immunotherapy for CRC be truly realized.

**Table 1:** Characteristics and applications of nanocarriers in colorectal cancer therapy.

Nanocarrier type	Key materials	Core advantages	Specific targets	Limitation & challenge	References
Liposomal Nanomedicines	DSPE-PEG, Hyaluronic Acid, PEGylated lipids	Prolonged half-life, passive targeting, active targeting via ligand conjugation, reduced systemic toxicity	Irinotecan+thalidomide, oxaliplatin, 5-FU+cannabidiol, anti-EGFR antibodies	Heterogeneity of EPR effect, poor tumor penetration, batch-to-batch variability	(27, 49–54)
Polymeric Nanoparticles	PLGA, PCL, PEG, PEGylated PLGA	Controlled drug release, improved drug solubility, prolonged colon retention, surface functionalization, co-delivery of multiple agents	Gambogenic acid, bevacizumab, paclitaxel + anti-PD-L1+anti-VEGF, silibinin, β-carotene + 5-FU	Potential for polymer-induced acidic byproducts; complexity of multi-component scale-up production.	(57–64)
Metal Nanoparticles	Branched gold nanoparticles, iron oxide nanoparticles, mesoporous silica-coated gold nanorods, gold nanocages	High photothermal/magnetothermal conversion efficiency, induction of ICD, multifunctional platform for combined therapy	CpG ODN + siPD-L1, anti-guanylyl cyclase monoclonal antibody, photothermal agents	Long-term toxicity, immunogenicity, accumulation concerns	(65–68)
Biomimetic and Natural-Based NPs	Platelet Membrane, E. coli Hybrids, Self-assembled TCM, HSA	Immune evasion; Natural tumor tropism; Protein-corona free; Complex metabolic regulation.	Defactinib; Porphyrin/E.coli; Wnt inhibitor; Silybin	Immunogenicity of biological components; Standardization of biological source extraction/purification; Complex manufacturing pipeline.	(5, 22, 27, 72, 74, 75, 77, 105)

## 4 Synergistic mechanisms of nanodrug delivery systems and immunotherapy

### 4.1 Improving tumor penetration of immune checkpoint inhibitors

MSS-type CRC is not sensitive to immune checkpoint inhibitors, and effective treatment regimens for this subtype of patients are currently under exploration (35). Nanocarrier technology offers innovative solutions to this problem. The developed MDPAs nanoplatform utilizes platelet-mimetic adhesion to achieve tumor accumulation in CRC (72). Structurally, this biomimetic platform consists of mesoporous silica nanoparticles preloaded with defactinib, which are further cloaked with platelet membranes functionalized with anti-PD-L1 antibodies. Benefiting from platelet-mediated tumor tropism, MDPAs can accumulate at tumor sites and suppress the integrin-FAK-YAP mechanical signaling cascade; blocking this axis relieves dense tumor stromal barriers and facilitates the intratumoral infiltration of cytotoxic T cells. Beyond elevating intratumoral drug concentration, the strategy reshapes the TME and consequently boosts combinatorial immunotherapeutic efficacy.

To achieve more precise drug release, nanoparticles are designed to respond to specific stimuli including acidic pH and ROS in the TME, ensuring precise drug release at the tumor site (73). For example, a pH and ROS dual-responsive autocatalytic release system (TPDM/PGA) was constructed to enhance CRC immunotherapy. This system dissociates in the acidic TME, promoting deep penetration and cellular uptake. Subsequently, under stimulation by high levels of H_2_O_2_, it releases drugs, generating abundant ROS, forming a positive feedback loop to accelerate drug release and exacerbate oxidative stress, thereby synergistically reversing the immunosuppressive TME (73). *In vivo* assays in CRC-bearing mice verified that this smart delivery platform substantially potentiates anti-PD-L1 treatment, slowing tumor progression and prolonging animal survival. Collectively, these preclinical outcomes highlight that nanotechnology-based drug delivery strategies, by improving the tumor penetration and controlled release of ICIs, provide powerful tools for overcoming resistance to immunotherapy in CRC, particularly MSS-type CRC.

### 4.2 Remodeling the immunosuppressive microenvironment

The immunosuppressive microenvironment of MSS-CRC is a core reason for its resistance to ICIs. Leveraging their unique delivery advantages, nanomedicines can precisely deliver immunostimulatory factors or drugs that inhibit immunosuppressive cells, thereby converting “cold” tumors into immunologically active “hot” tumors and enhancing the efficacy of subsequent immunotherapy. An apoptotic body membrane-coated ZIF nanoplatform co-loading ginsenoside Rg1 and atractylenolide I has been developed for colorectal cancer treatment (74). Furthermore, polymeric or albumin nanoparticles can deliver TGF-β inhibitors or Wnt/β-catenin pathway inhibitors, thereby reducing Treg infiltration and inhibiting their immunosuppressive function (75). Studies show that inhibiting the Wnt/β-catenin signaling pathway can enhance anti-tumor immune responses. A human serum albumin (HSA)-based nanodrug, GHSACA, achieves efficient targeted delivery via LOX1 receptor-mediated macropinocytosis. In MSS-CRC patient-derived xenograft (PDX) models, combining it with a PD-1 antibody resulted in a tumor growth inhibition (TGI) rate of up to 87.9%, significantly increased infiltration of CD3+/CD8+ CTLs, and reduced CD4+/CD25+ Tregs (75). Consistent with these findings, a ZIF nanoplatform co-delivering ginsenoside Rg1 and atractylenolide-I (Att) can promote DC maturation and enhance the expression of MHC class I molecules on tumor cells, thereby synergistically enhancing CTL infiltration and tumor recognition, increasing the tumor inhibition rate of PD-1 therapy from approximately 5% to 69% (74).

In combination therapy strategies, using nanomedicine to first remodel the microenvironment followed by administration of ICIs has proven effective in improving the immune response rate in MSS-CRC. For example, inhibiting the expression of the autophagy-related gene ATG16L1 can enhance the sensitivity of tumor cells to IFN-γ and TNF-mediated cell death, thereby increasing susceptibility to host immunity and creating favorable conditions for subsequent ICI therapy (76). Beyond nanomaterial-based modulation, synergistic regimens integrating oncolytic viruses with anti-PD-1 treatment also exert robust anti-tumor efficacy in MSS-CRC models. Such combinatorial treatment activates the innate immune system and upregulates pattern recognition receptors and antigen presentation-related markers, while reducing immunosuppressive macrophage populations and expanding functional effector T cells, thereby suppressing tumor progression (77). These studies indicate that nanomedicine-mediated microenvironment remodeling strategies, by synergistically modulating multiple immunosuppressive mechanisms, can overcome the resistance of MSS-CRC to immunotherapy, providing a solid theoretical foundation for clinical translation.

### 4.3 Induction of immunogenic cell death

ICD is a unique form of cell death characterized by the release or exposure of a series of damage-associated molecular patterns (DAMPs) from dying cells, thereby activating the host immune system and converting “cold” tumors into “hot” tumors. In CRC treatment, inducing ICD has become a key strategy to overcome the immunosuppressive TME and enhance immunotherapy efficacy. Various chemotherapeutic agents have been identified as effective ICD inducers. For example, first-line chemotherapeutics such as oxaliplatin can prompt CRC cells to expose calreticulin (CRT) on their surface and release DAMPs such as adenosine triphosphate (ATP) and high mobility group box 1 (HMGB1) (78). Furthermore, the novel topoisomerase I inhibitor lipotecan (TLC388) has been found to induce ICD, releasing HMGB1, ANXA1, and CRT, thereby remodeling the TME and enhancing the efficacy of radiotherapy (79). Besides chemotherapy, physical therapies like photothermal therapy (PTT) can also effectively induce ICD. Gold nanocage-mediated PTT in CRC models not only directly kills tumor cells but also induces immunogenic cell death and activates antigen-presenting cells, initiating anti-tumor immune responses (80).

NDDS show great potential in amplifying the ICD effect. By co-delivering ICD inducers with immune adjuvants, nanoplatforms can achieve spatiotemporally controlled drug release and synergistically enhance immunogenic signals. For example, an olsalazine-based metal-organic framework (MOF) nanocarrier was designed to co-deliver doxorubicin (DOX). Through an autocatalytic H_2_O_2_ regeneration mechanism, it enhances tumor-selective cytotoxicity and, combined with photothermal/chemodynamic therapy, effectively activates DAMP signals, amplifying the ICD effect (81). Another study developed folic acid-modified liposomes (FOS-lip) for co-delivering schisandrin A (SCHA) and oxaliplatin. By actively targeting CRC cells, these liposomes significantly elevate intracellular ROS levels, thereby enhancing oxaliplatin-induced ICD, promoting DAMP release and DC maturation (82). Furthermore, a MIL-100(Fe)-based nanoplatform (CRMH NPs) co-delivering CPT-11 and the immune agonist resiquimod (R848) achieves controlled release in the acidic TME, inducing ICD and activating the cGAS-STING pathway, synergistically enhancing chemo-immunotherapy effects (83).

**Figure 3:**
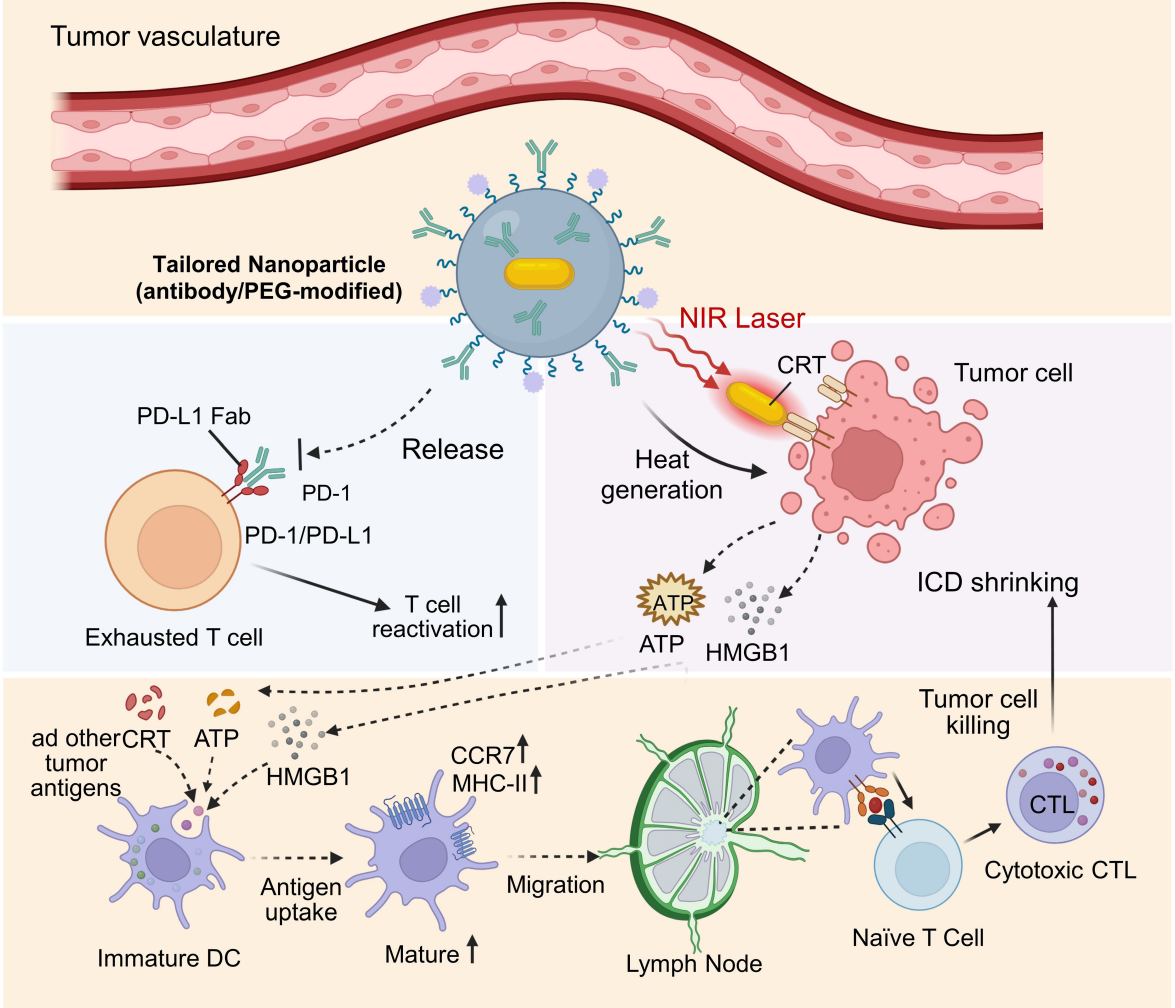
Schematic of the mechanism of nanoplatform remodeling of colorectal cancer tumor immune microenvironment. The TME is characterized by infiltration of immunosuppressive cells (Tregs, M2-TAMs, MDSCs), upregulation of immune checkpoints and impaired DC maturation and T cell priming. Nanomedicine delivery systems deliver immunomodulators to reprogram TAMs from M2 to M1, inhibit Tregs, and enhance DC maturation, thereby converting the immunosuppressive “cold” tumor into an immune-active “hot” tumor. (Image was created with BioRender.com.)

Preclinical studies provide strong evidence for the use of nanomedicines to induce ICD in the treatment of CRC. One study constructed multifunctional nanoparticles (OIMH NPs) by loading oxaliplatin and indocyanine green (ICG) into hyaluronic acid-modified MIL-100(Fe). Guided by photoacoustic imaging, this nanosystem induced ICD via a combined chemotherapy-photothermal therapy approach, enhanced T cell infiltration, and subsequently sensitized tumors to ICIs (anti-PD-L1), ultimately achieving significant tumor suppression in CRC models (84). Furthermore, a dual-targeting delivery system based on iron oxide nanoparticles (GOx@FeNPs) induced ICD by mediating PTT and ferroptosis. When combined with aPD-L1 ICIs, it achieved a tumor suppression rate exceeding 90% in CRC models (85). These studies collectively demonstrate that the precise delivery of ICD inducers via nanomedicines can effectively amplify immunogenic signals, remodel the tumor immune microenvironment, and provide a promising new strategy for CRC immunotherapy (**Figure 3**).

## 5 Preclinical advances in combination therapy strategies

### 5.1 Nanomedicine combined with immune checkpoint inhibitors

In the treatment of CRC, the efficacy of ICIs is often limited by the immunosuppressive nature of the TME. Nanomedicine delivery systems, capable of improving drug bioavailability, enabling targeted delivery, and modulating the immune microenvironment, offer novel strategies to enhance the efficacy of ICIs. In CT26 and MC38 CRC mouse models, multiple studies have explored the combined application of nanotechnology and ICIs. A study utilized PLGA nanoparticles to deliver anti-PD-L1 antibodies. The results showed that, compared to free antibodies, these nanoparticles not only prolonged the circulation time of antibodies *in vivo* but also enhanced anti-tumor immune responses and effectively suppressed MC38 tumor growth (86). Additionally, the co-delivery of chemotherapeutic agents with ICIs has shown synergistic effects. A study developed CD44v6-targeting PLGA nanoparticles loaded with irinotecan and combined them with anti-PD-L1 antibodies. In MC38 tumor-bearing mice, this combination therapy significantly reduced tumor burden, increased intratumoral infiltration of CD4+ and CD8+ T cells, and avoided systemic inflammatory responses (87). Beyond direct antibody delivery, nanosystems can also be used to deliver immune agonists to activate innate immune pathways. For instance, a CuS/MnO_2_ bimetallic nanosystem (diAMP-BCM) loaded with a STING agonist (e.g., cGAMP) was designed to preferentially activate type I interferon-mediated anti-tumor signaling. In MC38 and CT26 models, this nanosystem combined with anti-PD-1 therapy not only effectively inhibited primary tumor growth but also significantly enhanced the therapeutic effect on distant tumors by reshaping the T cell repertoire and alleviating T cell exhaustion, achieving a reversal of the “cold” TME (88). These studies collectively highlight the crucial role of nanomedicines in improving the efficacy of ICIs (**Table 2**).

**Table 2:** Summary of recent preclinical studies on nanomedicine combined with immunotherapy for mss colorectal cancer.

Nanoplatform	Payloads	Mechanism of action	Combination partner	In vivo model	Key finding
MDPAs (biomimetic nanodecoys)	Defactinib (FAK inhibitor) + PD-L1 antibody-conjugated platelet membrane	Inhibits integrin-FAK-YAP mechanotransduction, alleviates tumor mechanical barrier, promotes CTL infiltration	Anti-PD-L1	CT26 mouse model	Enhanced tumor accumulation, improved T cell infiltration, potentiated checkpoint blockade (72)
TPDM/PGA (pH/ROS dual-responsive system)	Autocatalytic drug release system	Dissociates in acidic TME, generates ROS upon H2O2 stimulation, accelerates drug release, reverses immunosuppressive TME	Anti-PD-L1	CRC mouse model	Improved therapeutic effect of anti-PD-L1, inhibited tumor growth, prolonged survival (73)
Apoptotic body membrane-coated ZIF nanoplatform	Ginsenoside Rg1 + Atractylenolide I (Att)	Promotes DC maturation, enhances MHC-I expression on tumor cells, increases CTL infiltration and tumor recognition	Anti-PD-1	MSS CRC model	Increased tumor inhibition rate from ~5% to ~69%, synergistic enhancement of PD-1 therapy (74)
GHSACA (HSA-based nanodrug)	LOX1-targeted therapeutic	LOX1 receptor-mediated macropinocytosis for efficient delivery, downregulates Wnt/β-catenin, reduces Tregs	Anti-PD-1	MSS CRC PDX model	TGI rate up to 87.9%, increased CD3+/CD8+ CTLs, reduced CD4+/CD25+ Tregs (75)
OIMH NPs (MOF-based)	Oxaliplatin + Indocyanine green (ICG)	Combined chemo-photothermal therapy, induces ICD, enhances T cell infiltration	Anti-PD-L1	CT26 mouse model	Significant tumor suppression, sensitized tumors to ICIs, photoacoustic imaging-guided (84)
GOx@FeNPs (iron oxide nanoparticles)	Glucose oxidase (GOx)-loaded Fe_3_O_4_ nanoparticles	Mediates PTT and ferroptosis, induces ICD, activates anti-tumor immunity	Anti-PD-L1	CRC mouse model	Tumor suppression rate >90%, promising synergistic effect with ICIs (85)
PLGA nanoparticles (targeted)	Irinotecan + CD44v6-targeting	Enhances tumor response to PD-L1 blockade, increases intratumoral CD4+/CD8+ T cells	Anti-PD-L1	MC38 tumor-bearing mice	Reduced tumor burden, avoided systemic inflammatory responses (87)
diAMP-BCM (CuS/MnO2 bimetallic nanosystem)	STING agonist (e.g., cGAMP)	Activates type I interferon-mediated anti-tumor inflammatory signaling, reshapes T cell repertoire, alleviates T cell exhaustion	Anti-PD-1	MC38 and CT26 models	Inhibited primary tumor growth, enhanced distant tumor effect, reversed “cold” TME (88)

Despite the significant potential shown by nanomedicines combined with ICIs in preclinical models, optimizing dosing and treatment timing to avoid immune-related adverse events remains a key challenge in clinical translation. For instance, the integration of chemoradiotherapy with dual immune checkpoint blockade targeting CTLA-4 and PD-1 has demonstrated synergistic antitumor efficacy and induction of durable T-cell immunity (89). Therefore, future research needs to more precisely design the release kinetics and targeting of nanomedicines to achieve spatiotemporal control of immune modulation, thereby maximizing efficacy while minimizing systemic immune toxicity.

### 5.2 Nanomedicine combined with CAR-T cell therapy

The combined application of nanomedicine and CAR-T cell therapy provides innovative strategies to overcome key obstacles in the treatment of solid tumors, including CRC. CAR-T cells face challenges in CRC treatment, including insufficient tumor infiltration, poor *in vivo* persistence, and an immunosuppressive TME (37) (90). Leveraging their programmable drug delivery properties, nanomedicines can precisely deliver immunomodulatory factors to enhance CAR-T cell function. For example, polymer nanoparticles can be designed to deliver γ-chain cytokines such as IL-15 or IL-21, which are key signaling molecules for maintaining T cell memory and effector functions. Cytokine combination therapy is a promising strategy to improve CAR-T therapy for CRC (91). Concurrently, nanocarriers such as liposomes can deliver chemokines such as CCL19. CEA CAR-T cells secreting XCL1 can mediate long-term anti-tumor immunity by enhancing the response of endogenous CD8+ T cells to tumor neoantigens (92).

Furthermore, nanomedicines demonstrate potential in remodeling the TME to enhance CAR-T cell efficacy. The immunosuppressive TME of CRC is a challenge for CAR-T cell therapy (39) (93). Studies have shown that in CRC xenograft models, delivering TGF-β inhibitors via nanomedicines can effectively reduce the physical and immune exclusion of CAR-T cells by the tumor stroma. Through local enrichment and controlled release by nanoparticles, TGF-β inhibitors can reach effective concentrations at the tumor site, block the TGF-β signaling pathway, thereby lifting the inhibition on T cell function and remodeling the tumor stroma. Experimental results indicate that CAR-T cells secreting PD-1-TREM2 scFv have stronger tumor clearance efficacy compared with CAR-T cells secreting PD-1 scFv (94). Dual chimeric antigen receptor T cells targeting PD-L1 and cancer-associated fibroblasts have shown *in vitro* efficacy in CRC (95).

Despite the promising prospects, the combination of nanomedicine and CAR-T cell therapy still faces numerous challenges. EpCAM CAR-T cells combined with Wnt inhibitor therapy for colorectal cancer can regulate the tumor microenvironment, improve T cell infiltration, promote effector T cell function and delay CAR-T cell exhaustion (96). Cytokine release syndrome (CRS) is one of the challenges faced in CAR-T therapy for colorectal cancer (97). Inappropriate dosing or timing of cytokines or immune agonists delivered by nanomedicines may exacerbate the risk of CRS. Therefore, there is a need to develop intelligent responsive nanocarriers that, for example, utilize specific enzymes or pH values in the TME to trigger drug release, thus achieving more precise regulation.

### 5.3 Nanomedicine combined with tumor vaccines

The strategy of combining nanomedicine with tumor vaccines shows great potential in CRC treatment. Its core lies in utilizing nanocarriers to efficiently deliver antigens or immune-stimulating molecules to activate specific anti-tumor immune responses. LNPs, as a representative platform in this field, have been successfully used for the co-delivery of mRNA vaccines encoding neoantigens and the Toll-like receptor 4 (TLR4) agonist monophosphoryl lipid A (MPLA). Polymeric nanoparticles and lipid nanoparticles are both important nanodelivery systems for targeted therapy of CRC (58) (98). LNPs co-delivering miR159 mimics and irinotecan have pH-sensitive properties that enable endosomal escape for the treatment of CRC (99).

Polymer nanoparticles also have unique advantages in simplifying vaccine preparation processes. Polymer nanoparticles have good biocompatibility and anti-cancer properties in 3D co-culture sphere models of CRC (100). Targeted nanocarriers have broad application prospects in the diagnosis, treatment, and theranostics of CRC (101). For example, PLGA-based nanoparticles loaded with 5-fluorouracil have been confirmed to efficiently enhance the therapeutic effect in CRC (102). Additionally, polymer nanoparticles-based oral drug delivery systems have made progress in the treatment of CRC (103).

Emerging preclinical evidence further validates that combinatorial regimens integrating nanovaccines with ICIs are capable of breaking immune tolerance in CRC. Targeted lipid nanotherapies against VPS9D1-AS1 have been shown to sensitize CRC tumors to PD-1 blockade therapy, substantially improving the overall efficacy of immune checkpoint immunotherapy (104). In addition, engineered Lactobacillus reuteri modified with nanomaterials can mediate photodynamic immunotherapy of CRC and regulate tryptophan metabolism of gut microbiota (105). Specifically, this nano-modified Lactobacillus reuteri can exert synergistic immunotherapeutic efficacy through the combined effect of phototherapy and regulation of gut microbiota metabolites, adding a new dimension to the combination strategy (105). These findings suggest that the combination of nanomedicine and tumor vaccines is a key strategy for achieving breakthroughs in immunotherapy through multi-target, multi-pathway synergistic effects.

## 6 Challenges and solutions in clinical translation

### 6.1 Biosafety of nanomedicines

While nanomedicines show great promise in CRC treatment, their clinical translation faces challenges related to biosafety, particularly long-term toxicity and immunogenicity.

Metal nanoparticle accumulation, for instance, can induce neuroinflammation, though PEGylation appears to mitigate this risk. Immunogenicity, particularly the generation of anti-PEG antibodies upon repeated administration, poses an equally critical challenge, as it accelerates drug clearance and may trigger hypersensitivity. While alternative surface modifications and PEG chain conformation optimization offer partial solutions, the long-term safety and efficacy of these substitutes remain poorly characterized, constituting a significant impediment to clinical translation. A more viable strategy lies in coupling rigorous physicochemical standardization with individualized assessment of host immune responses. Ultimately, overcoming the biosafety hurdles of nanomedicines requires not only advanced biomaterials but also surface design tailored to individual patients.

### 6.2 Tumor heterogeneity and personalized treatment

The heterogeneity of CRC is one of the core challenges in its treatment. This heterogeneity is reflected not only in molecular subtypes but also across multiple levels including microsatellite status, gene mutation profiles, and immune characteristics of the TME, directly leading to significant differences in patient responses to immunotherapy and nanomedicines. Therefore, developing precise biomarkers is a key prerequisite for achieving personalized treatment. BRAF V600E-mutant CRC can be further divided into two transcriptional subtypes, BM1 and BM2. The BM1 subtype is characterized by heightened immune reactivity, whereas the BM2 subtype exhibits prominent activation of cell cycle-related gene signatures. Clinical studies have confirmed that patients with the BM1 subtype receiving combined treatment with dabrafenib, trametinib, and panitumumab had significantly better progression-free survival and overall survival than those with the BM2 subtype, indicating that transcriptional background can serve as an independent predictive biomarker beyond mutation status alone (106). Additionally, mutations in the ARID1A gene are associated with higher tumor mutational burden and survival benefit after immune checkpoint inhibitor treatment in various cancers. However, in CRC, its association with immune infiltration is cancer type-dependent, suggesting that its potential as a pan-cancer predictive marker needs to be interpreted within the context of specific cancer types (107). Furthermore, the Consensus Molecular Subtype classification, particularly the CMS4 subtype, is associated with stromal infiltration and poorer prognosis, while high expression of CD14 is associated with the CMS4 subtype, BRAF mutation, and right-sided CRC, and predicts worse clinical outcomes. These molecular and immunological signatures provide novel stratification criteria to refine personalized immunotherapeutic strategies for CRC patients (108, 109).

Liquid biopsy technology, especially the detection of circulating tumor DNA (ctDNA), provides a non-invasive, dynamic tool for real-time monitoring of tumor genetic evolution and immune status, which is crucial for dose adjustment of nanomedicines and the formulation of combination therapy strategies. In anti-EGFR therapy, liquid biopsy has been used to assess resistance mechanisms. The CAPRI-GOIM study aims to evaluate the role of liquid biopsy and explore potential new resistance mechanisms to anti-EGFR therapy (110). Furthermore, a case report described that a patient with MSI metastatic colorectal cancer complicated by autoimmune disease still responded to immune checkpoint inhibitor therapy during steroid treatment (111). In the field of nanomedicines, monitoring changes in mutation abundance in ctDNA can allow for early judgment of the therapeutic effect of nanocarrier drug delivery systems and timely adjustment of dosing regimens, achieving dynamic personalized treatment. Delivery of siRNA targeting the primate-specific long non-coding RNA FLANC via nanoparticles reduces metastasis in colorectal cancer mouse models, and FLANC has potential as a biomarker and therapeutic target (112). In summary, personalized nanovaccines and targeted ligand design are key to improving the survival outcomes of CRC patients.

## 7 Latest research achievements and cutting-edge technologies

### 7.1 Intelligent responsive nanoplatforms

Intelligent responsive nanoplatforms represent a significant breakthrough in the field of targeted immunotherapy for CRC in recent years. Their core advantage lies in their ability to sense and utilize the unique physicochemical characteristics of the TME, such as low pH, high ROS levels, and overexpression of specific enzymes, thereby achieving precise drug release and local enrichment. This design strategy significantly improves the bioavailability of therapeutic drugs while minimizing systemic toxicity to normal tissues. pH-responsive nanoparticles designed for the acidic microenvironment of CRC can specifically release PD-L1 targeting miRNA, VCP/p97 inhibitors and TLR7 agonists at the tumor site, achieving preferential tumor accumulation and minimizing off-target exposure (113). This spatially controlled release mechanism not only enhances the efficacy of ICIs but also improves their safety, offering a new approach to overcome the systemic side effects of traditional immunotherapy.

In addition to pH responsiveness, enzyme-responsive nanoplatforms also show great potential in CRC treatment. Overexpressed matrix metalloproteinases (MMPs) in the tumor stroma can serve as endogenous triggers. Enzyme-responsive nanoparticles can improve the targeted delivery and anti-tumor efficacy of eugenol in human CRC cells, promoting apoptosis and inhibiting migration and invasion (114). This strategy can effectively improve drug distribution within the tumor and promote the infiltration of immune cells, transforming immune “cold” tumors into immune “hot” tumors (115). Furthermore, targeting characteristics such as high glutathione (GSH) and hydrogen sulfide (H_2_S) expression in CRC, researchers have developed dual-responsive nanoplatforms. For instance, F127MOF-199 nanoparticles dissociate in the GSH-rich TME to release copper ions inducing cuproptosis, while simultaneously converting into Cu_2_S nanoparticles in the H_2_S environment, triggering pyroptosis via photothermal/chemodynamic therapy, synergistically activating a powerful anti-tumor immune response (116). These studies demonstrate that intelligent responsive nanoplatforms integrate TME-responsive release with multimodal immune regulation to provide translational strategies for the precise immunotherapy of CRC.

### 7.2 Multimodal imaging-guided combination therapy

By incorporating multiple imaging functionalities such as fluorescence imaging, magnetic resonance imaging (MRI), and photoacoustic imaging (PAI), nanomedicines can provide high-resolution, high-sensitivity anatomical and molecular information, thereby guiding the optimization of treatment decisions. Magnetically mediated magnetothermal therapy combined with CSF1R inhibitor can be used for immunotherapy of “cold” CRC (117). One study constructed a theranostic nanomedicine based on manganese dioxide (MnO_2_) (MnO_2_@MPDA-PEG NPs). This nanoplatform responsively releases Mn^2+^ in the TME, which promotes the immune response induced by mild PTT, including DC maturation, thereby effectively enhancing systemic anti-tumor immunity in CT26 CRC models (118).

**Figure 4:**
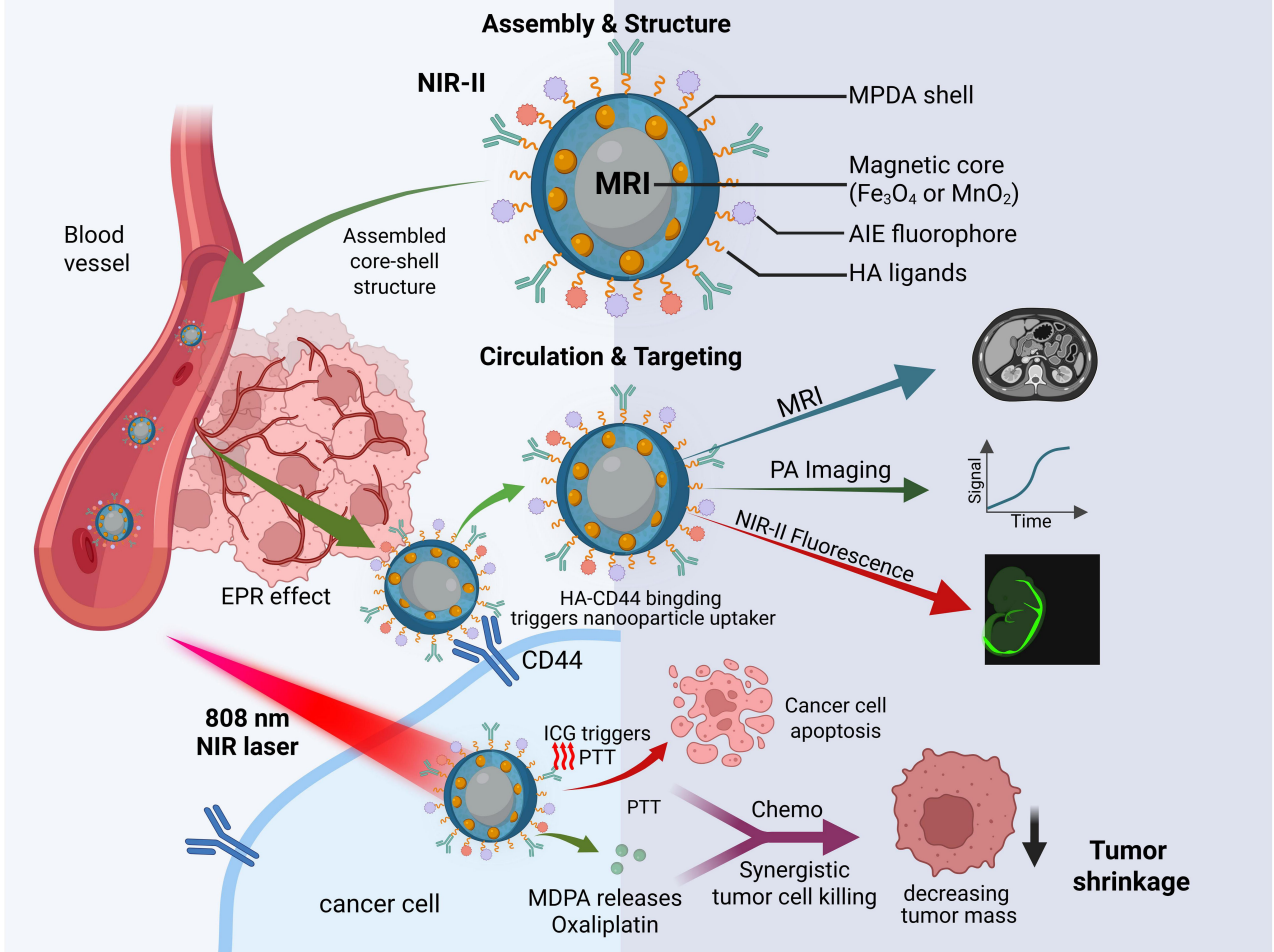
Mechanisms of nanomedicine in enhancing checkpoint blockade and inducing immunogenic cell death. (1) Nanocarriers co-deliver anti-PD-L1 antibodies and chemotherapy to overcome stromal barriers and improve drug penetration. (2) Stimuli-responsive nanoparticles (pH/ROS/enzyme-sensitive) release TGF-β inhibitors or TLR agonists to modulate the TME. (3) Specific nanoprobes such as metal-organic frameworks (MOFs) or gold nanocages enable photothermal or chemodynamic therapy, inducing ICD by exposing calreticulin (CRT), releasing ATP and HMGB1. (Image was created with BioRender.com).

Through real-time imaging feedback, clinicians can dynamically adjust treatment plans, achieving personalized precision therapy. The study by Zhu et al. utilized an aggregation-induced emission (AIE) material combined with E. coli to construct a biomimetic robot (EcN@INX-2), achieving near-infrared-II (NIR-II) fluorescence imaging-guided photodynamic/photothermal combination therapy with immunotherapy. This platform can achieve multimodal imaging and treatment in CRC models *in vivo*, supporting image-guided combined immunotherapy (119). Furthermore, a multifunctional nanoparticle (OIMH NP) based on a metal-organic framework (MOF) was able to co-load a chemotherapy drug (oxaliplatin) and a photosensitizer (indocyanine green). Through PAI-guided chemo-photothermal combination therapy, it effectively induced ICD and activated T cells, subsequently enhancing sensitivity to ICIs (81). This imaging-guided combination therapy strategy not only improves treatment precision but can also predict treatment response and resistance by early monitoring changes in the TME, such as the density of TREM2+ TAMs, allowing for timely adjustment of treatment strategies (120). In summary, multimodal imaging-guided combination therapy, by integrating diagnostic and therapeutic functions, provides a powerful tool for precision medicine in CRC (**Figure 4**).

## 8 Future research directions and prospects

### 8.1 Optimization of multi-target combination strategies

Future research should focus on exploring strategies where nanomedicines simultaneously target multiple immune checkpoints or immune pathways to overcome the issue of resistance in single-target therapies. The introduction of artificial intelligence (AI) and machine learning technologies provides powerful tools for optimizing multi-target combination strategies. By analyzing vast amounts of genomic, proteomic, and clinical data, AI algorithms can predict optimal drug combinations, dose ratios, and treatment timing, significantly reducing the cost and time of traditional experimental screening. A risk prediction model based on glycosyltransferase constructed using transcriptomic data can effectively predict CRC patient responses to ICIs, providing a basis for formulating personalized combination therapy regimens (121). Similarly, a risk score model based on hypoxia and lipid metabolism-related genes also demonstrates predictive value for the prognosis and immunotherapy sensitivity of CRC patients (122). These models can be integrated into AI-driven decision support systems to predict the efficacy of different multi-target combinations in specific patient subgroups, thereby achieving true precision medicine. Furthermore, machine learning can assist in designing the physicochemical properties of nanomedicines, optimizing their *in vivo* delivery efficiency, and ensuring that multiple therapeutic payloads are delivered synchronously and efficiently to the tumor site.

### 8.2 Clinical translation pathway for nanomedicines

For nanomedicines to transition from the laboratory to clinical application, simplifying manufacturing processes and establishing standardized quality control are primary key priorities. In recent years, microfluidic technology has garnered significant attention due to its ability to precisely control fluid mixing and enable continuous production. In the development of curcumin (CUR)-loaded P123:F127:TPGS polymeric micelles (PFT: CUR), researchers successfully prepared a nanosystem with uniform particle size (approximately 15.9 nm) by optimizing the hydration temperature in the thin-film hydration method, although this process still requires fine control. In contrast, microfluidic technology can synthesize nanoparticles with controlled size and narrow distribution in a one-step process by adjusting flow rates and channel geometry, significantly improving manufacturing efficiency and reproducibility. Additionally, establishing a standardized quality control system is crucial. This includes rigorous testing of key physicochemical properties of nanomedicines such as particle size, polydispersity index (PDI), zeta potential, drug loading, encapsulation efficiency, and *in vitro* release behavior. For instance, one study synthesized HA-mPEG-Cis nanoparticles with an average hydrodynamic diameter of 48 nm and a polydispersity index of 0.13 (123). Only by simplifying processes and establishing unified quality standards can a solid production foundation be laid for the clinical translation of nanomedicines.

### 8.3 Development of personalized nano-immunotherapies

The core of personalized nano-immunotherapy lies in utilizing the patient’s own tumor genomic and immunomic data to design patient-specific nanomedicines for precision immunotherapy. This strategy first relies on comprehensive molecular subtyping of the patient’s tumor. Analyzing ligand-receptor interactions within the TME via single-cell RNA sequencing can identify ligand-receptor pairs that affect the abundance of tumor-infiltrating lymphocytes and potential therapeutic targets (124). Based on this information, nanocarriers that can specifically target tumor cells can be designed. For instance, targeting EpCAM which is overexpressed in CRC, researchers have developed corresponding aptamer-modified nanoparticles for delivering birch triterpenoid analogs, thereby enhancing efficacy while reducing off-target toxicity to normal cells (125). Furthermore, small molecule drug conjugates targeting the PI3K/mTOR pathway and pH-responsive RNA nanoparticles delivering PI3K/mTOR inhibitors have been developed for the treatment of CRC (126, 127). This nanotechnology-based personalized vaccine platform offers new ideas to overcome the challenge of low immunogenicity in CRC.

**Figure 5:**
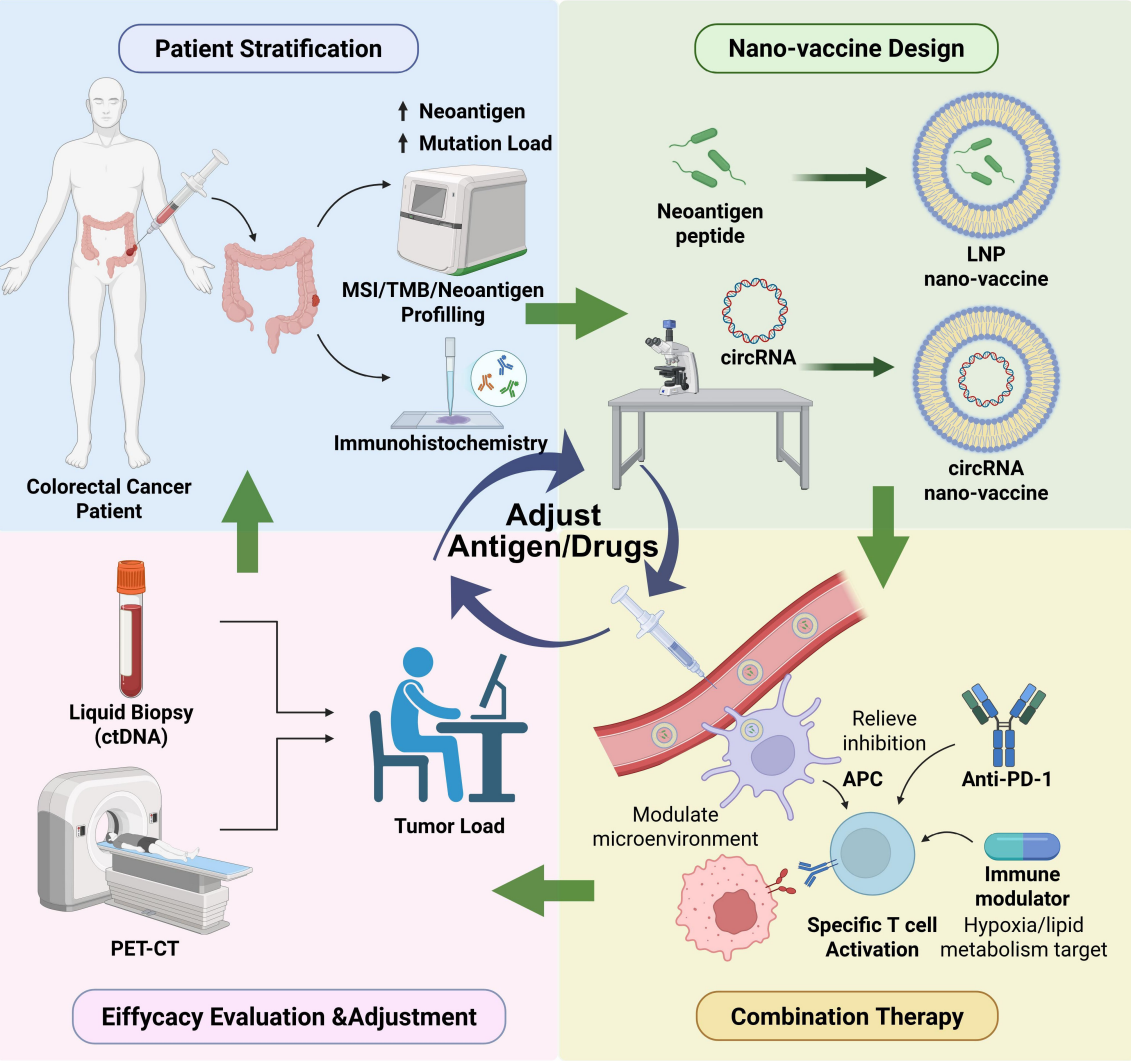
Nanotechnology-based personalized immunotherapy strategies for colorectal cancer. (1) Patient stratification: tumor biopsy, genetic sequencing, and immunohistochemical analysis are performed to identify tumor mutational burden and neoantigens in CRC patients. (2) Nanovaccine design: patient-specific neoantigen loaded nanovaccines are designed and synthesized according to the sequencing results. (3) Combination therapy: nanovaccines are combined with immune checkpoint inhibitors or other immunomodulatory agents. (4) Therapeutic evaluation and adjustment: therapeutic effects are dynamically evaluated by liquid biopsy and imaging. Based on the evaluation results, the combination regimen is adjusted to achieve individualized precision immunotherapy. (Image was created with BioRender.com).

Despite the promising prospects of personalized nano-immunotherapy, its clinical translation still faces significant ethical and cost challenges. High research and development costs, as well as complex individualized manufacturing processes, limit its broad accessibility. To address these issues, multi-faceted efforts such as policy support, insurance coverage, and the development of standardized nanomedicine platforms are needed to promote the development of personalized treatments (**Figure 5**).

## 9 Conclusion

The synergistic integration of targeted immunotherapy and nanomedicines opens new avenues for CRC treatment. Nanocarriers not only address the issues of low delivery efficiency and poor tumor penetration of traditional immunotherapeutic drugs but also achieve immune activation of “cold” tumors by remodeling the immunosuppressive microenvironment and inducing ICD. Preclinical evidence shows that this strategy can significantly enhance anti-tumor immune responses in both MSI-H and MSS-type CRC models, indicating potential across molecular subtypes. Clinical translation of these nanomedicine approaches for MSS-type CRC has yet to be fully established and requires rigorous validation in future clinical trials.

Moreover, clinical translation currently faces several core challenges, including the lack of unified standards for biosafety assessment of nanomaterials, individual response differences caused by tumor heterogeneity, and the complexity of managing immune-related toxicities. Future research should focus on multi-target combination strategies, personalized nanomedicine design based on patient tumor immune profiles, and optimization of clinical translation pathways to further improve the prognosis and quality of life of CRC patients.

## Glossary

ACKR4: Atypical chemokine receptor 4
AI: Artificial intelligence
AIE: Aggregation-induced emission
ATP: Adenosine triphosphate
CAR-T: Chimeric antigen receptor T cell
CBD: Cannabidiol
CD: Cluster of differentiation
CRC: Colorectal cancer
CRT: Calreticulin
CTLA-4: Cytotoxic T-lymphocyte-associated protein 4
CTLs: Cytotoxic T lymphocytes
DAMPs: Damage-associated molecular patterns
DCs: Dendritic cells
DCR: Disease control rate
dMMR: Deficient mismatch repair
EGFR: Epidermal growth factor receptor
EPR: Enhanced permeability and retention
FDA: U.S. Food and Drug Administration
GSH: Glutathione
HA: Hyaluronic acid
HER2: Human epidermal growth factor receptor 2
HMGB1: High mobility group box 1
HSA: Human serum albumin
ICD: Immunogenic cell death
ICG: Indocyanine green
ICIs: Immune checkpoint inhibitors
LAG-3: Lymphocyte activation gene 3
LNPs: Lipid nanoparticles
MC38: Murine colon carcinoma cell line
mCRC: Metastatic colorectal cancer
MDSCs: Myeloid-derived suppressor cells
MHC-I: Major histocompatibility complex class I
MMPs: Matrix metalloproteinases
MOFs: Metal-organic frameworks
MRI: Magnetic resonance imaging
MSI: Microsatellite instability
MSS: Microsatellite stable
MUC1: Mucin 1
NDDS: Nanodrug delivery systems
NIR: Near-infrared
NPs: Nanoparticles
ORR: Objective response rate
PAI: Photoacoustic imaging
PD-1: Programmed death receptor 1
PD-L1: Programmed death-ligand 1
PDX: Patient-derived xenograft
PEG: Polyethylene glycol
PFS: Progression-free survival
PLGA: Poly (lactic-co-glycolic acid)
pMMR: Proficient mismatch repair
PTT: Photothermal therapy
ROS: Reactive oxygen species
STING: Stimulator of interferon genes
TAMs: Tumor-associated macrophages
TGI: Tumor growth inhibition
TIGIT: T cell immunoglobulin and ITIM domain
TIM-3: T-cell immunoglobulin and mucin domain-containing molecule 3
TME: Tumor microenvironment
TMB: Tumor mutational burden
Tregs: Regulatory T cells
VEGF: Vascular endothelial growth factor
